# Supplementary material for: Assembly and seasonality of core phyllosphere microbiota on perennial biofuel crops
Source: Nat Commun. 2019 Sep 12;10:4135. doi: 10.1038/s41467-019-11974-4 (PMC6742659; doi:10.1038/s41467-019-11974-4)
Supplement: Supplementary file 1 — Supplementary Information [file 41467_2019_11974_MOESM1_ESM.pdf]

Supplementary Figure 1

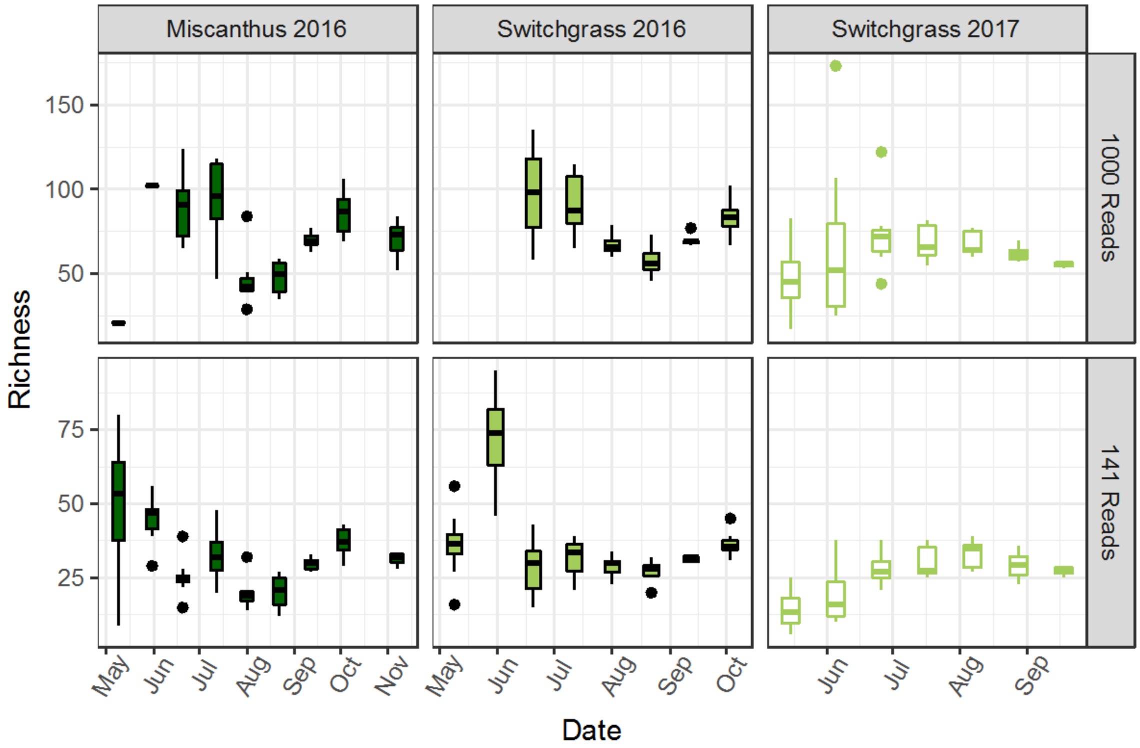

Seasonal patterns in the number of observed phyllosphere taxa (richness). Operational taxonomic units (OTUs) were defined at 97% amplicon sequence identity. Richness is provided at subsampling depths of 1000 reads (top) and 141 reads (bottom). Whiskers show maximum and minimum values, excluding outliers.

Supplementary Figure 2

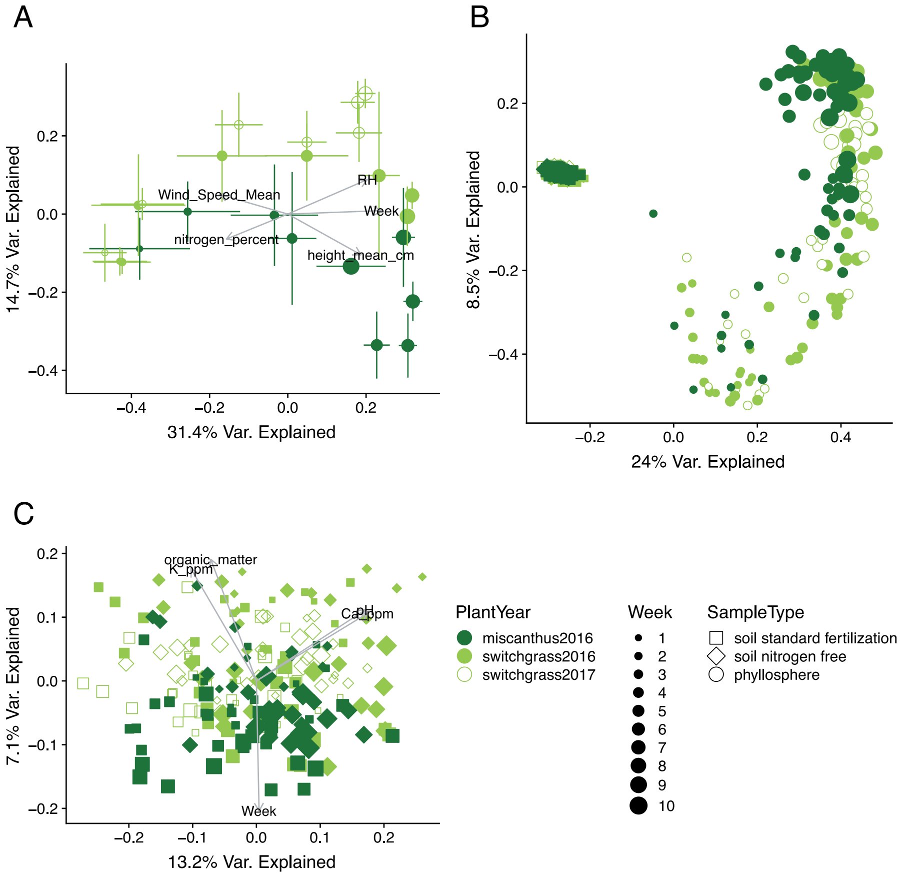

Seasonal patterns in the structures of bacterial and archaeal communities inhabiting the phyllosphere and associated soils of the biofuel feedstocks switchgrass and miscanthus. (A) Principal coordinates analysis (PCoA) of switchgrass and miscanthus phyllosphere communities (Bray-Curtis dissimilarity), error bars show 1 deviation around the centroid (n = 3 to 8 replicate plots/time point). Subsampling depth was 141 reads per sample and environmental vectors are fitted when $r^2 > 0.4$ and $p < 0.05$. (B) PCoA of the phyllosphere communities relative to the soil, subsampled to 141 sequences per sample. (C) PCoA of the soil communities associated with miscanthus and switchgrass, subsampled to 19,967 sequences per sample.

Supplementary Figure 3

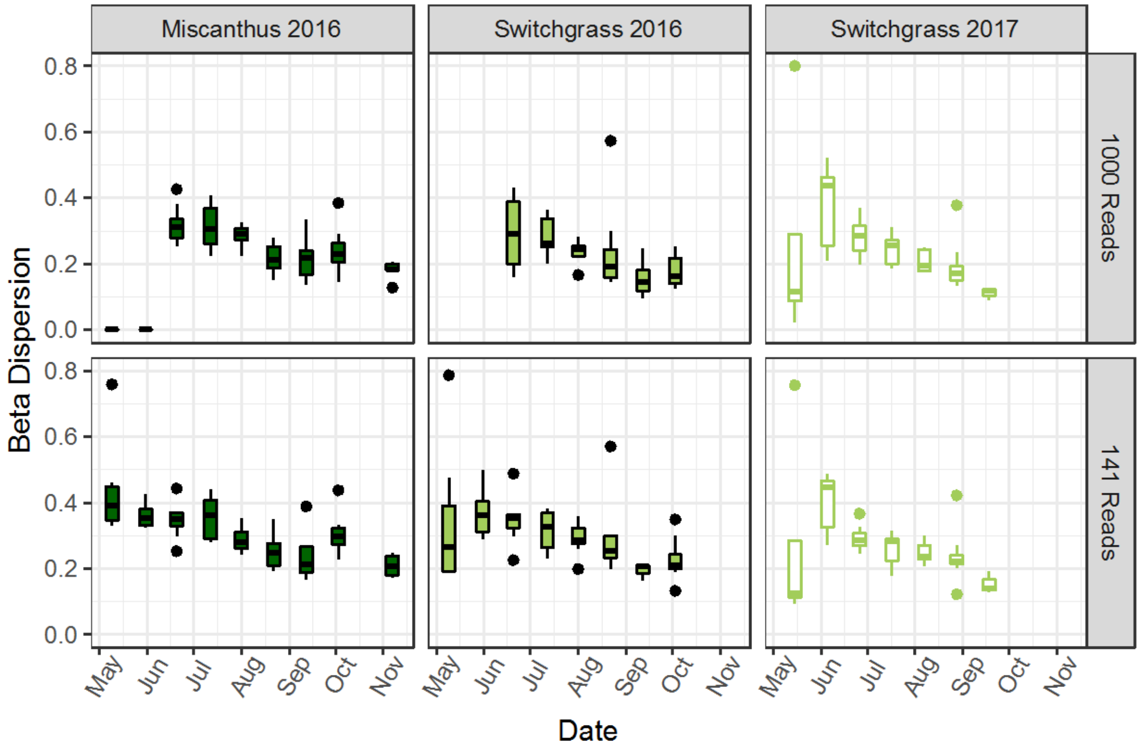

Phyllosphere communities become less variable over time. Distance to median was calculated by analysis of beta-dispersion. Variability in phyllosphere microbiome structure over time miscanthus 2016, switchgrass 2016, and switchgrass 2017 field seasons. Betadispersion was calculated from data series subsampled to 1000 reads (top) and 141 reads (bottom). Whiskers show maximum and minimum values, excluding outliers.

Supplementary Figure 4

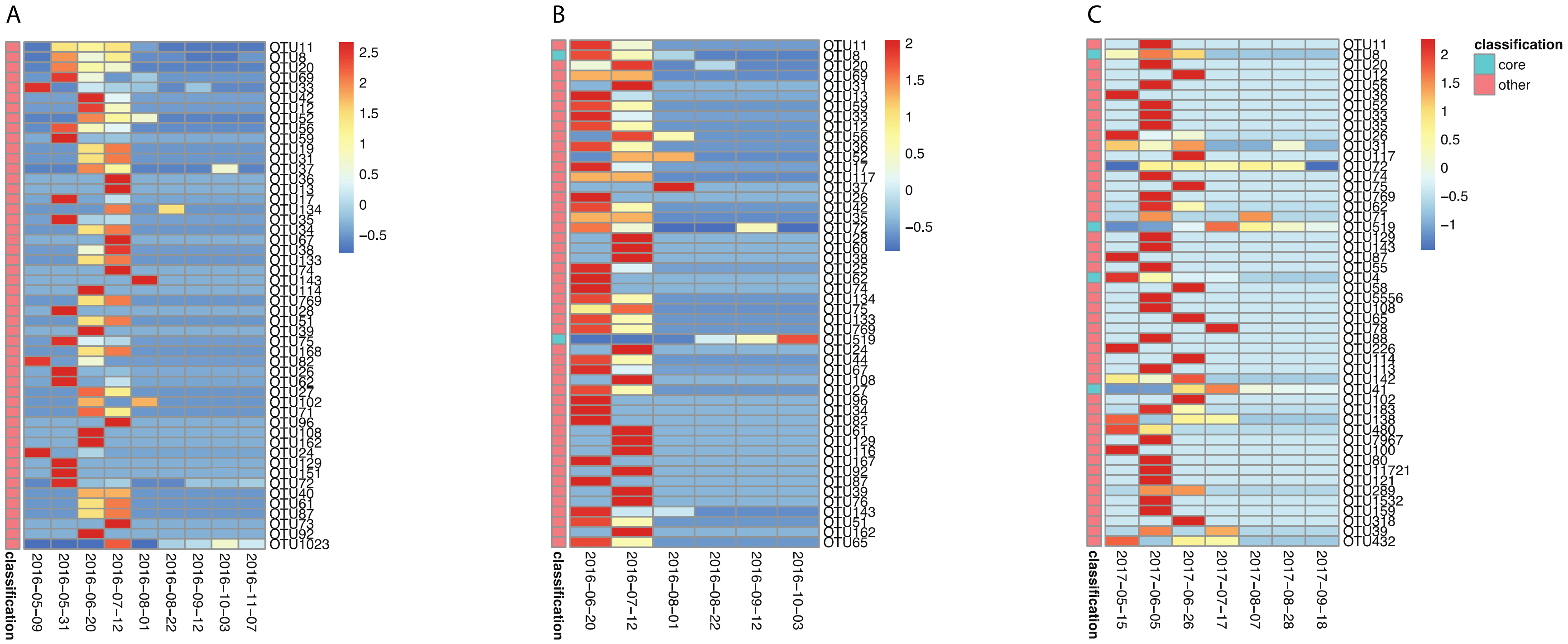

Decreases in the contributions of soil-dominating taxa to the phyllosphere microbiome over time. Heatmaps represent the 50 top-ranked OTUs from the soil that were also detected in the phyllosphere. The cell colors are the z-scored relative abundances of the OTUs in the phyllosphere. The ranking is from top to bottom (e.g., the most abundant soil-dominant taxon that was also detected in the phyllosphere is represented by the top row in the heatmap). The left bar shows the classification the taxon as either a core member (green) or not (pink). **(A)** Miscanthus 2016; **(B)** switchgrass 2016; **(C)** switchgrass 2017.

Supplementary Figure 5

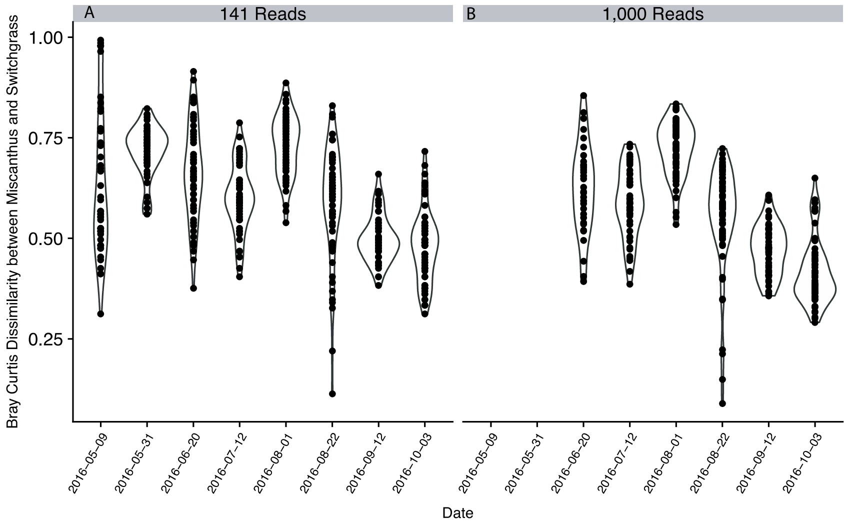

Bray Curtis Dissimilarity between Miscanthus and Switchgrass over time in 2016. We show changes in Bray-Curtis dissimilarities between switchgrass and miscanthus phyllosphere microbiomes per time point, inclusive of the maximum number of replicated blocks (up to 8) per time point. **(A)** “Full” time series, subsampled to 141 reads per sample. **(B)** Time series subsampled to 1000 reads per sample. The area of the violins is constant for each time point.

Supplementary Figure 6

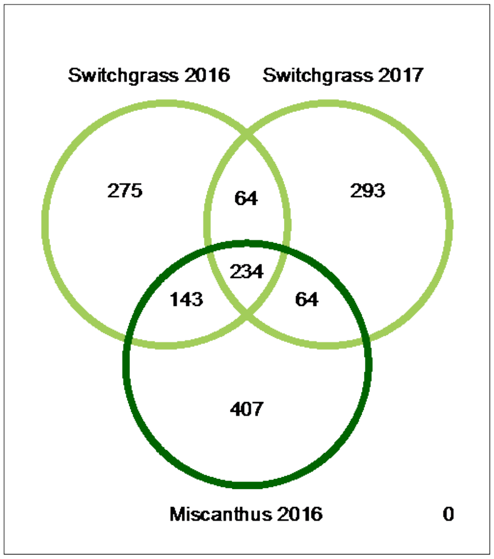

Venn diagram of taxa shared across the switchgrass and miscanthus phyllosphere, in 2016 and 2017. Data were rarefied to 1000 reads per sample.

Supplementary Figure 7

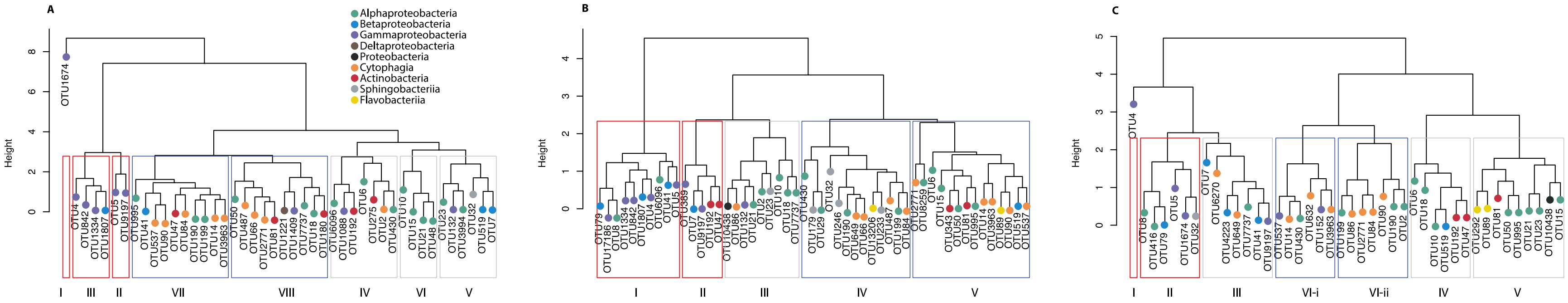

Hierarchical clustering of standardized (z-scored) dynamics of core phyllosphere taxa on the phyllosphere of miscanthus 2016 (A), switchgrass 2016 (B) and switchgrass 2017 (C). Seasonally discrete clusters coincide with plant phenology, including groups that achieved highest relative abundance during early (red), mid (gray) and late (blue) plant growth. Clusters are labeled in order of temporal occurrence in **Figure 4G-I**. Circles on the dendrogram tips are color coded by OTU taxonomic classification and labeled with the OTU ID.

	Miscanthus 2016	Switchgrass 2016	Switchgrass 2017
Raw Read Pairs	7336923	7120682	9271169
QC Reads	7142900	6883631	7123783
% Chloroplast/ Mitochondria of QC Reads	77.27%	67.16%	12.32%
Samples >= 141 Reads	65	62	44
Samples >= 1,000 Reads	53	43	44
Samples >= 10,000 Reads	36	27	44
OTUs (rarefied to 141 Reads)	540	685	232
OTUs (rarefied to 1,000 Reads)	1010	859	769
OTUs (rarefied to 10,000 Reads)	1121	896	2320
**Number of samples per sampling week (dataset rarefied to 1000 reads)**	Phyllosphere | soil Plots (1, 2, 3, 4)		
1	NA |(2,1,2,1)	NA |(1,2,2,2)	NA |(2,2,2,2)
2	(1,0,0,0)|(2,1,2,2)	(0,0,0,0)|(2,1,2,2)	(1,2,1,0)|(2,1,2,2)
3	(0,0,1,0)|(2,1,2,2)	(0,0,0,0)|(2,1,2,2)	(2,1,2,2)|(2,1,2,2)
4	(2,2,2,2)|(2,1,2,2)	(2,1,0,1)|(2,1,2,2)	(1,2,2,2)|(2,2,2,2)
5	(2,2,0,2)|(2,1,2,2)	(2,2,2,2)|(2,1,2,2)	(2,2,2,2)|(2,2,2,2)
6	(2,2,2,2)|(2,2,2,2)	(2,2,2,2)|(2,2,1,2)	(2,2,1,2)|(2,2,2,2)
7	(2,2,2,2)|(2,2,2,2)	(2,2,2,2)|(2,2,2,2)	(2,2,2,2)|(2,2,2,2)
8	(2,2,2,1)|(2,2,2,2)	(2,2,2,1)|(2,2,2,2)	(1,0,1,1)|(2,2,2,2)
9	(2,2,2,2)|(2,2,2,2)	(2,2,2,2)|(2,2,2,2)	NA | NA
10	(1,1,2,2)|(2,2,2,2)	NA | NA	NA | NA

Supplementary Table 1

Sequencing summary of phyllosphere microbial communities characterized in this study, categorized by crop and year. QC is quality controlled and NA is not applicable (due to inter-annual seasonal variation in crop phenology).

Supplementary Table 2

	141 Reads	500 Reads	1000 Reads	5000 Reads
500 Reads	0.932			
1000 Reads	0.872	0.982		
5000 Reads	0.752	0.931	0.976	
10000 Reads	0.725	0.916	0.967	0.999

Comparison of overarching patterns of beta diversity across the same dataset rarefied to different sequencing depths. We compared all pairs of 141, 500, 1000, 5000, or 10000 reads per sample. All Mantel tests were significant at $p < 0.001$ on 1000 permutations.

Supplementary Table 3

Variable Tested	Axis 1	Axis 2	R squared	P -value
precipitation	-0.127	0.146	0.038	0.703
Air_temp_mean	-0.298	-0.078	0.095	0.384
air_temp_max	-0.212	-0.056	0.048	0.633
Air_Temp_Min	-0.242	0.139	0.078	0.439
Air_Pressure	-0.022	0.217	0.048	0.653
Relative Humidity	0.474	0.444	0.422	0.008
Actual Humidity	-0.081	0.102	0.017	0.834
Wind_Speed_Mean	-0.703	-0.213	0.539	0.001
Solar_Radiation	-0.258	-0.368	0.202	0.118
PAR	-0.300	-0.288	0.173	0.162
soil_temp_5_cm_bare_avg	-0.408	-0.061	0.170	0.163
Week	0.730	0.356	0.659	0.001
Leaf dry matter content_mg_per_g	0.418	0.177	0.206	0.001
nitrogen_percent	-0.392	-0.416	0.327	0.001
carbon_percent	0.345	0.038	0.121	0.001
carbon_per_nitrogen	0.274	0.134	0.093	0.003
Leaf height_mean_cm	0.730	-0.230	0.586	0.001
pH	-0.027	0.219	0.049	0.047
P_ppm	-0.120	-0.097	0.024	0.187
K_ppm	-0.445	0.187	0.233	0.001
Ca_ppm	0.047	0.059	0.006	0.659
Mg_ppm	-0.366	-0.125	0.149	0.001
organic_matter	-0.348	-0.036	0.123	0.001
NO3N_ppm	0.003	-0.080	0.006	0.646
NH4_ppm	-0.461	0.090	0.220	0.001
soil_moisture_percent	0.609	0.015	0.371	0.001
soil_temp_10cm	-0.364	-0.028	0.133	0.001

Fitted environmental variables that explain changes in microbiome community structure. Values in which EnvFit $R^2 > 0.40$ were plotted as vectors in **Figure 2.**
