# Supplementary material for: Assembly and seasonality of core phyllosphere microbiota on perennial biofuel crops
Source: Nat Commun. 2019 Sep 12;10:4135. doi: 10.1038/s41467-019-11974-4 (PMC6742659; doi:10.1038/s41467-019-11974-4)
Supplement: Supplementary file 3 — Reporting Summary [file 41467_2019_11974_MOESM3_ESM.pdf]

nature research

Corresponding author(s): Shade, Ashley

Last updated by author(s): Jul 23, 2019

Reporting Summary

Nature Research wishes to improve the reproducibility of the work that we publish. This form provides structure for consistency and transparency in reporting. For further information on Nature Research policies, see Authors & Referees and the Editorial Policy Checklist.

Statistics

For all statistical analyses, confirm that the following items are present in the figure legend, table legend, main text, or Methods section.

n/a	Confirmed	
☐	☒	The exact sample size (*n*) for each experimental group/condition, given as a discrete number and unit of measurement
☐	☒	A statement on whether measurements were taken from distinct samples or whether the same sample was measured repeatedly
☐	☒	The statistical test(s) used AND whether they are one- or two-sided<br>*Only common tests should be described solely by name; describe more complex techniques in the Methods section.*
☐	☒	A description of all covariates tested
☐	☒	A description of any assumptions or corrections, such as tests of normality and adjustment for multiple comparisons
☐	☒	A full description of the statistical parameters including central tendency (e.g. means) or other basic estimates (e.g. regression coefficient) AND variation (e.g. standard deviation) or associated estimates of uncertainty (e.g. confidence intervals)
☐	☒	For null hypothesis testing, the test statistic (e.g. *F*, *t*, *r*) with confidence intervals, effect sizes, degrees of freedom and *P* value noted<br>*Give P values as exact values whenever suitable.*
☒	☐	For Bayesian analysis, information on the choice of priors and Markov chain Monte Carlo settings
☒	☐	For hierarchical and complex designs, identification of the appropriate level for tests and full reporting of outcomes
☐	☒	Estimates of effect sizes (e.g. Cohen's *d*, Pearson's *r*), indicating how they were calculated

Our web collection on statistics for biologists contains articles on many of the points above.

Software and code

Policy information about availability of computer code

Data collection	These sequencing data were generated with the Illumina platform. Base calling was doing using RTA v1.18.54 from the raw data, according to the Department of Energy's Joint Genome Institute standard operating protocols.
Data analysis	cutadapt v1.17 was used from removing adapters and primers from paired reads.<br>usearch v10.0.240_i86linux64 was used for read merging, quality filtering, otu clustering and classification.<br>R v3.4.0 was used for data analysis.<br>cowplot v0.9.4 was used for combining plots into a single figure.<br>dplyr v0.7.6 was used for manipulating data structures.<br>ggplot2 v3.1.0 was used for data visualization.<br>limma v3.32.10 was used for venn diagrams.<br>pheatmap v1.0.12 was used for producing heatmap.<br>reshape2 v1.4.3 was used for manipulating data structures.<br>RSQLite v2.1.1 was used for accessing SQLite databases.<br>scales v1.0.0 was used for formatting dates.<br>tidyr v0.8.1 was used for data formatting.<br>vegan v2.5-4 was used for ecological statistics.

For manuscripts utilizing custom algorithms or software that are central to the research but not yet described in published literature, software must be made available to editors/reviewers. We strongly encourage code deposition in a community repository (e.g. GitHub). See the Nature Research guidelines for submitting code & software for further information.

Data

Policy information about availability of data

All manuscripts must include a data availability statement. This statement should provide the following information, where applicable:

- Accession codes, unique identifiers, or web links for publicly available datasets
- A list of figures that have associated raw data
- A description of any restrictions on data availability

Analysis workflows used to produce all figures are available on GitHub (ShadeLab/PAPER_GradySorensenStopnisek_InPrep)
Amplicon sequences used to produce all figures are available through JGI, project IDs 1191521 and 1163036.

Field-specific reporting

Please select the one below that is the best fit for your research. If you are not sure, read the appropriate sections before making your selection.

☐ Life sciences ☐ Behavioural & social sciences ☒ Ecological, evolutionary & environmental sciences

For a reference copy of the document with all sections, see nature.com/documents/nr-reporting-summary-flat.pdf

Ecological, evolutionary & environmental sciences study design

All studies must disclose on these points even when the disclosure is negative.

Study description	This work is an observational study, conducted at the Kellogg Biological Station's (KBS) Biofuel Cropping System Experiment (BCSE) which was established in 2008 using a randomized complete block design.
Research sample	Samples were collected at 3 flags distributed across each plot and subplot, then pooled to represent one sample. Soil samples consist of three 2x10 mm cores, pooled and homogenized together. Leaf samples consist of 10 leaves collected at each flag, pooled and stored together.
Sampling strategy	Samples were chosen based off the BCSE complete randomized block design, to incorporate both the nitrogen fertilized main plot and the unfertilized sub-plots.
Data collection	Environmental data was collected from a variety of measures including on-site (soil temperature, air temperature) collected by the researchers, published weather data collected by the MSU Enviro-weather Automated Weather Station Network at the KBS weather station, soil and leaf chemistry were measured at MSU's Soil and Plant Nutrient Laboratory. Amplicon libraries were created and sequencing data was collected by the Department of Energy's Joint Genome Institute.
Timing and spatial scale	Two crop types - switchgrass and miscanthus were sampled every 3 wees from pre-emergence (bare soil only) through senescence (when >50% of plants are brown and dry) in 2016. For the 2017 growing season, we focused on switchgrass as it has been identified as a DOE priority biofuel crop. We selected three-week sampling intervals to capture a full phenological We sampled replicates 1-4, both the 30 x 40 m main plot as well as the 3 x 40 m unfertilized subplots. Distance between sampling sites ranged from 55.4 to 406.2 m between centroid points.
Data exclusions	Samples were excluded from certain analyses when they contained fewer than 1000 OTUs, to provide higher overall sequencing depth, though we present supplemental data containing analyses rarefied at both 1000 OTUs and the minimum, 146 OTUs for full transparency. For clarity, the rarefaction number has been listed for every figure.
Reproducibility	All analysis workflow scripts are readily available, to facilitate transparency and reproducibility. Because this is an observational study over multiple growing seasons, experimental replication is not possible. As this is an observational study, repeated experimental attempts are not applicable.
Randomization	Sample sites were randomized in the original BCSE experimental design. As our study is conducted over time and must account for seasonal variations and the changing phenology of the host plant, sample randomization or blinded analysis was not appropriate.
Blinding	Sample blinding was not relevant in this observational study, as sampling time was an important component of our analyses.

Did the study involve field work? ☒ Yes ☐ No

Field work, collection and transport

Field conditions	Field conditions including precipitation, soil moisture, and soil temperature were recorded at each time of sampling and were included in our metadata and analyses. Our relevant contextual data for this study are available on GitHub (ShadeLab/ PAPER_GradySorensenStopnisek_InPrep)

Location	All sampling for this study was conducted at the Kellogg Biological Station, on the Great Lakes Bioenergy Research Center's Biofuel System Cropping Experiment study site, located in Hickory Corners, MI (42.395152, -85.373624).
Access and import/export	Sampling was conducted in accordance with the KBS and GLBRC policies, and under the permission of the Site Use Request Form entitled "Seasonal dynamics of switchgrass and miscanthus phyllosphere microbiota" for 2016 and 2017.
Disturbance	Site access and sampling was conducted in accordance with the GLBRC's standard walking path to minimize site disturbance. Cores were collected at designated sampling sites only.

Reporting for specific materials, systems and methods

We require information from authors about some types of materials, experimental systems and methods used in many studies. Here, indicate whether each material, system or method listed is relevant to your study. If you are not sure if a list item applies to your research, read the appropriate section before selecting a response.

Materials & experimental systems

n/a	Involved in the study
☒	☐ Antibodies
☒	☐ Eukaryotic cell lines
☒	☐ Palaeontology
☒	☐ Animals and other organisms
☒	☐ Human research participants
☒	☐ Clinical data

Methods

n/a	Involved in the study
☒	☐ ChIP-seq
☒	☐ Flow cytometry
☒	☐ MRI-based neuroimaging
