# Supplementary material for: Assembly and seasonality of core phyllosphere microbiota on perennial biofuel crops
Source: Nat Commun. 2019 Sep 12;10:4135. doi: 10.1038/s41467-019-11974-4 (PMC6742659; doi:10.1038/s41467-019-11974-4)
Supplement: Supplementary file 4 — Description of Additional Supplementary Files [file 41467_2019_11974_MOESM4_ESM.pdf]

Description of Additional Supplementary Files

File Name: Supplementary Data 1
Description: Operational taxonomic unit identifiers and representative 16S rRNA gene amplicon sequences of the core switchgrass and miscanthus phyllosphere microbiota.
